# Brain region‐specific accumulation of amyloidosis‐associated proteins in postmortem brain tissues of Alzheimer's disease patients

**DOI:** 10.1002/alz70855_104912

**Published:** 2025-12-24

**Authors:** Wangchen Tsering, Jennifer L. Phillips, Jonathan Anthony B. Villareal, Stefan Prokop

**Affiliations:** ^1^ University of Florida, Gainesville, FL, USA; ^2^ University of Florida / Center for Translational Research in Neurodegenerative Disease, Gainesville, FL, USA; ^3^ University of Florida / Department of Pathology, Gainesville, FL, USA; ^4^ University of Florida / Fixel Institute for Neurological Diseases, Gainesville, FL, USA; ^5^ University of Florida / McKnight Brain Institute, Gainesville, FL, USA

## Abstract

**Background:**

The matrisome is a comprehensive list of molecules that form and interact with the extracellular matrix (ECM). The matrisome includes not only structural proteins but also signaling molecules and regulators that that can modify the ECM. Proteomic studies in human and animal models of Alzheimer's disease (AD) have shown alterations in matrisome proteins in AD. We previously showed that some of the matrisome proteins increase during the progression of AD and can modify Aβ plaques and cerebral amyloid angiopathy (CAA). However, the role of these proteins in AD pathogenesis and their characterization in postmortem brain tissue remain unclear.

**Method:**

We employed a detailed immunohistochemistry study on postmortem brain tissues representing different neuropathological changes, namely Low AD (*n* = 6), Intermediate AD (*n* = 6), and High AD cases (*n* = 6), to elucidate the accumulation of five matrisome proteins (MDK, SPOCK3, Col25a1, SDC4 and EGFL8) in four different brain regions (the occipital cortex, hippocampus, striatum, and cerebellum), and their association with Aβ plaques, neurofibrillary tangles (NFT), and CAA.

**Result:**

We found that all matrisome proteins increase with the disease progression in the occipital cortex and hippocampus. Interestingly, similar to Aβ plaque, MDK staining was observed in all brain regions examined, while SPOCK3, Col25a1, EGFL8, and SDC4 staining were rare in the striatum and completely absent in the cerebellum. SPOCK3 staining shows region‐specific vulnerability, with a significantly higher burden in the hippocampus compared to other brain regions. All matrisome proteins stain only a subset of Aβ plaques. The colocalization and staining patterns of matrisome proteins suggest that some of these proteins may mediate tau spreading and represent different phases of Aβ deposition.

**Conclusion:**

Overall, our results suggest that matrisome proteins can influence the Aβ deposition, serve as biomarkers for AD, and can offer a new avenue for developing drugs that target matrisome proteins.

